# The apatinib and pemetrexed combination has antitumor and antiangiogenic effects against NSCLC

**DOI:** 10.1515/biol-2022-0533

**Published:** 2023-03-07

**Authors:** Ling Zhou, Wenchao Zhang, Yi Xiang, Zijun Qian, Jianping Zhou, Lei Ni, Yun Feng, Beili Gao

**Affiliations:** Department of Respiratory Medicine, Rui Jin Hospital, Shanghai Jiao Tong University, School of Medicine, Shanghai 200025, China; Department of Allergy, Henan Provincial People’s Hospital, Henan University, Zhengzhou 450000, China

**Keywords:** apatinib, NSCLC, zebrafish model, pemetrexed

## Abstract

Chemotherapy for advanced non-small-cell lung cancer (NSCLC) remains the first treatment choice. Angiogenesis inhibitors are effective for lung cancer treatment. This study explored whether chemotherapy combined with angiogenesis inhibitors could achieve better efficacy in NSCLC. The zebrafish A549 xenograft model was used to investigate the combined effect of apatinib and chemotherapeutic agents in NSCLC. Apatinib combined with pemetrexed demonstrated the highest antitumor effect compared with apatinib combined with gemcitabine or paclitaxel *in vitro*. In the zebrafish A549 xenograft model, apatinib and pemetrexed, alone or in combination, showed significant inhibition of tumor growth. Co-treatment with apatinib and pemetrexed demonstrated the best antitumor effects, suggesting that the combination of apatinib and pemetrexed might be a promising alternative therapy for patients with lung cancer. Apatinib combined with pemetrexed had enhanced antitumor effects compared with either one alone in the zebrafish model of NSCLC.

## 1 Introduction

Lung cancer is one of the most frequent malignancies globally [1,2]. Chemotherapy is the mainstay of treatment for inoperable non-small-cell lung cancer (NSCLC) [3,4,5]. Effective cytotoxic drugs in lung cancer, as supported by guidelines, include gemcitabine, paclitaxel, and pemetrexed in combination with platinum salts [3,4,5]. Although chemotherapeutic drugs kill many tumor cells, the remaining tumor cells can continue to grow, participating in treatment failure or recurrences [6]. Angiogenesis also plays a critical role in cancer by providing nutrient and oxygen supplies to fast-growing tumors, and targeting angiogenesis has become a targeted therapy strategy [7]. These drugs inhibit the effect of the vascular endothelial growth factor (VEGF) on its receptor (VEGFR) and angiogenesis [8]. In addition, these drugs assist in the survival of tumor blood vessels, reduce the pressure between tumor tissues, improve the delivery of chemotherapy drugs to tumor tissues, and improve the chemotherapy effect [8].

Apatinib, also known as YN968D1, is a tyrosine kinase inhibitor that selectively inhibits VEGFR2 [9,10]. It is an orally bioavailable, small molecular agent that inhibits angiogenesis by inhibiting VEGF-mediated endothelial cell migration and proliferation, thus blocking the formation of new blood vessels in tumor tissues [9,10]. Apatinib is effective in patients with NSCLC after failure with other therapies [11,12], as supported by meta-analyses [13,14]. In addition, Apatinib might be useful in increasing the intracellular concentrations of antineoplastic agents in several chemotherapy-resistant cancer cell lines [9,10]. Apatinib is potentially effective when combined with conventional chemotherapeutic agents, especially in cases of resistance to chemotherapy [15].

The Dll4-Notch-hey2 pathway is a critical negative regulator of tumor angiogenesis, restraining excessive VEGF-induced vascular sprouting and angiogenesis. The expression of Dll4 depends on continuous VEGF signaling, but blockage of the VEGF signaling pathway causes a rapid and marked decrease in the expression of Dll4 in the tumor vessels. According to a study, blockage of Dll4 inhibited tumor growth by promoting non-productive angiogenesis [16,17]. The EFNB2A gene regulates the proper spatial activation of VEGFR2 endocytosis in zebrafish [18]. SLIT2 promotes angiogenic activity via the ROBO1-VEGFR2-ERK1/2 pathway [19], and the SLIT3-ROBO4 pathway promotes vascular network formation [20]. The fibroblast growth factor receptor (FGFR) signaling pathway is associated with vascular outgrowth and maintenance of blood vessel integrity [21].

This study explored whether chemotherapy combined with vascular inhibitors can achieve better efficacy in NSCLC.

## 2 Methods

### 2.1 Cell culture

The A549 cell line was obtained from the Shanghai Institutes for Biological Sciences (China) and cultured in F12 Nutrient Mix-Kaighns Mod (Invitrogen) medium supplemented with 10% fetal bovine serum (FBS; Invitrogen) and 1% antibiotic/antimycotic (Invitrogen) at 37°C in a humidified atmosphere containing 5% CO_2_.

### 2.2 Cell viability

A549 cells were seeded at 5×10^4^ cells/well in 96-well microculture plates and cultured for 12 h. The medium was removed and replaced with a fresh medium with or without cytotoxic agents, apatinib, and apatinib combined with cytotoxic agents. The cells were incubated for 72 h. The medium was removed and replaced with fresh medium. Cell survival was then quantified using the tetrazolium dye methylthiotetrazole assay (MTT). Each experiment was repeated at least thrice.

### 2.3 Combination index analyses

In this experiment, apatinib and cytotoxic agents (gemcitabine, paclitaxel, and pemetrexed) were used. These cytotoxic agents were selected because they are among the first-line treatment options for NSCLC [3,4,5]. Apatinib was combined with the cytotoxic agents in four proportions (4/5 apatinib + 1/5 cytotoxic agent, 3/5 + 2/5, 2/5 + 3/5, and 1/5 + 4/5). The initial concentration of each agent was chosen based on the IC_50_ value of these four combination proportions. For the combination of apatinib and gemcitabine, the IC_50_ value of the apatinib single-agent was 5.384 μM, and the IC_50_ value of the gemcitabine single-agent was 0.750 μM. We chose 4 μM apatinib + 1 μM gemcitabine, 3 μM apatinib + 2 μM gemcitabine, 2 μM apatinib + 3 μM gemcitabine, and 1 μM apatinib + 4 μM gemcitabine, as initial concentrations in the four combination modes. The cell viability of A549 cells in the apatinib + gemcitabine group was measured by the MTT assay. The final IC_50_ value of apatinib and that of gemcitabine in the mixture of apatinib + gemcitabine were determined according to the MTT assay. Similarly, in the combination of apatinib + paclitaxel, the IC_50_ value of the apatinib single-agent was 2.693 μM, and that of the paclitaxel single-agent was 0.408 nM. We used 4 μM apatinib + 0.4 nM paclitaxel, 3 μM apatinib + 0.8 nM paclitaxel, 2 μM apatinib + 1.2 nM paclitaxel, and 1 μM apatinib + 1.6 nM paclitaxel. In the combination of apatinib and pemetrexed, the IC_50_ value of the apatinib single-agent was 5.275 μM, and that of the pemetrexed single-agent was 0.876 μM. We used 4 μM apatinib + 1 μM pemetrexed, 3 μM apatinib + 2 μM pemetrexed, 2 μM apatinib + 3 μM pemetrexed, and 1 μM apatinib + 4 μM pemetrexed. The A549 cell viability in different treatment groups is presented in Supplementary Material. The IC_50_ values of apatinib and chemotherapy agents in different groups are presented in Supplementary Material.

### 2.4 Zebrafish

The zebrafish models were obtained from the Shanghai Research Center of the Southern model organisms. Adult zebrafish were maintained at 28.5°C on a 14 h light/10 h dark cycle. Five to six pairs of zebrafish were set up for mating, and 200–300 embryos were generated on average. The embryos were maintained at 28.5°C in fish water (0.2% instant ocean salt in deionized water). The embryos were washed and staged according to the Kimmel CB’s experience [22]. The zebrafish facility at Shanghai Research Center for Model Organisms is accredited by the Association for Assessment and Accreditation of Laboratory Animal Care (AAALAC) International. The study was approved by the Animal Care and Use Committee of the Shanghai Research Center of the Southern model organisms.


**Ethical approval:** The research related to animal use has been complied with all the relevant national regulations and institutional policies for the care and use of animals.

### 2.5 Maximum non-lethal concentration (MNLC)

In order to determine the MNLC and LC_50_ of the compounds, zebrafishes were exposed from 2 days post-fertilization (dpf) through 5 dpf to record the mortality. Dead zebrafishes were defined as those with an absence of heartbeat under a dissection stereomicroscope (Nikon SMZ645; Japan). For the initial tests, the compounds at five concentrations (0.1, 1, 10, 100, and 1,000 μM/L) were used for evaluation. If no MNLC and LC_50_ were found from the initial tests, additional concentrations of 0.01–2,000 μM/L were used for testing. Mortality curves were generated using GraphPad Prism 5.0 (GraphPad Software, San Diego, CA, USA), and MNLC was determined using logistic regression.

### 2.6 Angiogenesis

As the cells reach the horizontal myoseptum, the segmental artery tip cell undergoes a single cell division (20.5–23.5 hours post-fertilization (hpf)), and after this, one cell maintains its position and becomes a connector cell, while the tip cell continues to migrate dorsally, forming dorsal longitudinal anastomotic vessel (DLAV, 30–31 hpf). By 2 hpf, the trunk and tail intersegmental vessels (ISVs) for most zebrafish are lumenized and have an active circulation [23,24].

In order to evaluate the formation of blood vessels in zebrafish embryos, 22 hpf embryos were distributed in 12-well plates (30 embryos per well) (BD Falcon) for a treatment period of 26 h. The positive control for this assay was 5 μM PTK787, which is a VEGFR antagonist [25], and the negative control was 0.1% dimethyl sulfoxide (DMSO). Different concentrations of apatinib (1, 2.5, 5, and 10 μM) and PTK787 (5 μM) were diluted in 0.1% DMSO. DMSO containing the drug of interest was soaked in fish water, and zebrafish could take the drug orally. After treatment, the embryos were anesthetized with 0.016% MS-222 (tricaine methanesulfonate, Sigma-Aldrich, St. Louis, MO) and the number of complete ISVs (i.e., the number of ISVs that connect the dorsal aorta (DA) to the DLAV) was counted. The drug effect was then calculated using the following formula:

\% \text{Inhibition}=\left(1-\frac{\text{ISV amount of experimental group}}{\text{ISV amount of vehicle control}}\right)\times 100.]

### 2.7 Zebrafish tumor xenograft

Zebrafish embryos were obtained using standard mating conditions and staged for cell xenoplantation at 48 hpf. After cancer cell staining, the embryos were dechorionated using the micro-forceps, anesthetized with 0.0016% tricaine, and then positioned on their right side on a wet 1.0% agarose pad. The cells were stained with 5 μg/mL CM-DiI diluted in phosphate buffered saline (PBS) and washed four times: once with FBS, twice with PBS, and then once with 10% FBS diluted in PBS. The cells were kept on ice before injection. Approximately 200 cancer cells were injected into the yolk sac. The embryos with red fluorescence were moved to 6-well plates and cultured in fish water containing the drugs of interest (5 μmol apatinib with or without 70.5 μmol pemetrexed and 70.5 μmol pemetrexed). Tumor growth on day 4 of post-injection (dpi) was measured by fluorescence microscopy, and the relative fluorescence intensity (RFI) was measured to estimate the volume of tumor xenografts in control and drug-treated animals. Finally, the experiments were terminated, and the animals were euthanized by overexposing to tricaine.

### 2.8 Image acquisition

The embryos and larvae were analyzed with a Nikon SMZ 1500 Fluorescence microscope and photographed with digital cameras. Quantitative image analyses were performed using image-based morphometric analysis (NIS-Elements D3.1, Japan).

### 2.9 Quantitative real-time PCR

Total RNA was extracted from 30 to 50 embryos per group using Trizol (Roche) according to the manufacturer’s instructions. RNA was reverse transcribed using the PrimeScript RT reagent Kit with gDNA Eraser (Takara). Quantification of gene expression was performed in triplicates using Bio-rad iQ SYBR Green Supermix (Bio-rad) by detecting on the Realplex system (Eppendorf). The relative gene expression quantification was based on the comparative threshold cycle method (2^−ΔΔCt^) using *ef1* as the endogenous control gene. The primer sequences are provided in Supplementary Material. Three independent experiments were evaluated.

### 2.10 Statistical analysis

All data are presented as mean value ± SEM. Statistical analysis and graphical representation of the data were evaluated using GraphPad Prism 5.0 (GraphPad Software, San Diego, CA). Statistical significance was evaluated using Student’s *t*-test, analysis of variance (ANOVA) with the LSD post hoc test, or *χ* ^2^ test, as appropriate. *P <* 0.05 was considered statistically significant, and statistical significance was indicated by *P < 0.05, and ***P < 0.0001.

## 3 Results

### 3.1 Synergistic effects of apatinib combined with chemotherapy in A549 cell line

The MTT results showed that apatinib single-agent had a slight inhibitory effect on the viability of A549 lung cancer cells, with an IC_50_ value ranging from 2.693 to 5.384 μmol. The IC_50_ value of the apatinib single-agent was higher than that of the three cytotoxic agents (gemcitabine 0.75 μmol, paclitaxel 0.48 nmol, and pemetrexed 0.88 μmol), suggesting that apatinib had low cytotoxicity compared with chemotherapy agents (Supplementary Material).

**Figure 1:**
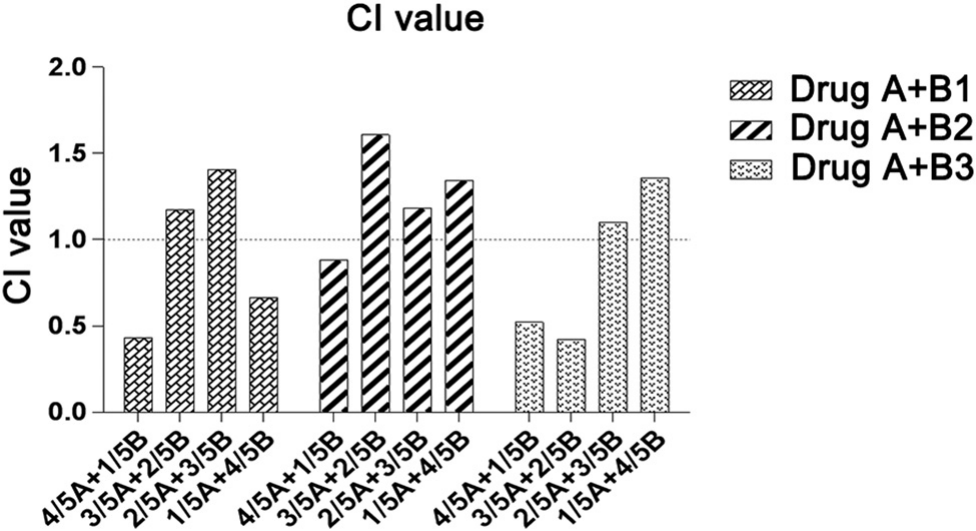
Combination index of apatinib (A) and gemcitabine (B1), paclitaxel (B2), and pemetrexed (B3) in A549 cell line. There are four combination proportions of apatinib and cytotoxic agents (from 1/5 A + 4/5 B to 4/5 A + 1/5 B). The combination index (CI) was used to estimate the combination effects.

The combination of apatinib and gemcitabine showed a synergistic effect at 4/5 apatinib + 1/5 gemcitabine (CI = 0.43) and 1/5 apatinib + 4/5 gemcitabine (CI = 0.67). In the combination of apatinib and paclitaxel, a synergistic effect at the proportion of 4/5 apatinib + 1/5 paclitaxel (CI = 0.88) was observed. In the combination of apatinib and pemetrexed, a synergistic effect at the proportion of 4/5 apatinib + 1/5 pemetrexed (CI = 0.52) and 3/5 apatinib + 2/5 pemetrexed (CI = 0.42) was observed (Figure 1). These results suggested that apatinib combined with pemetrexed at the proportion of 3/5 apatinib + 2/5 pemetrexed (CI = 0.42) provided an optimal antitumor effect *in vitro*. These findings suggest that apatinib combined with the three cytotoxic agents provides a synergistic antitumor effect at rational combination proportions, especially apatinib and pemetrexed.

### 3.2 Toxicity and safety of apatinib or pemetrexed in the zebrafish model

**Figure 2:**
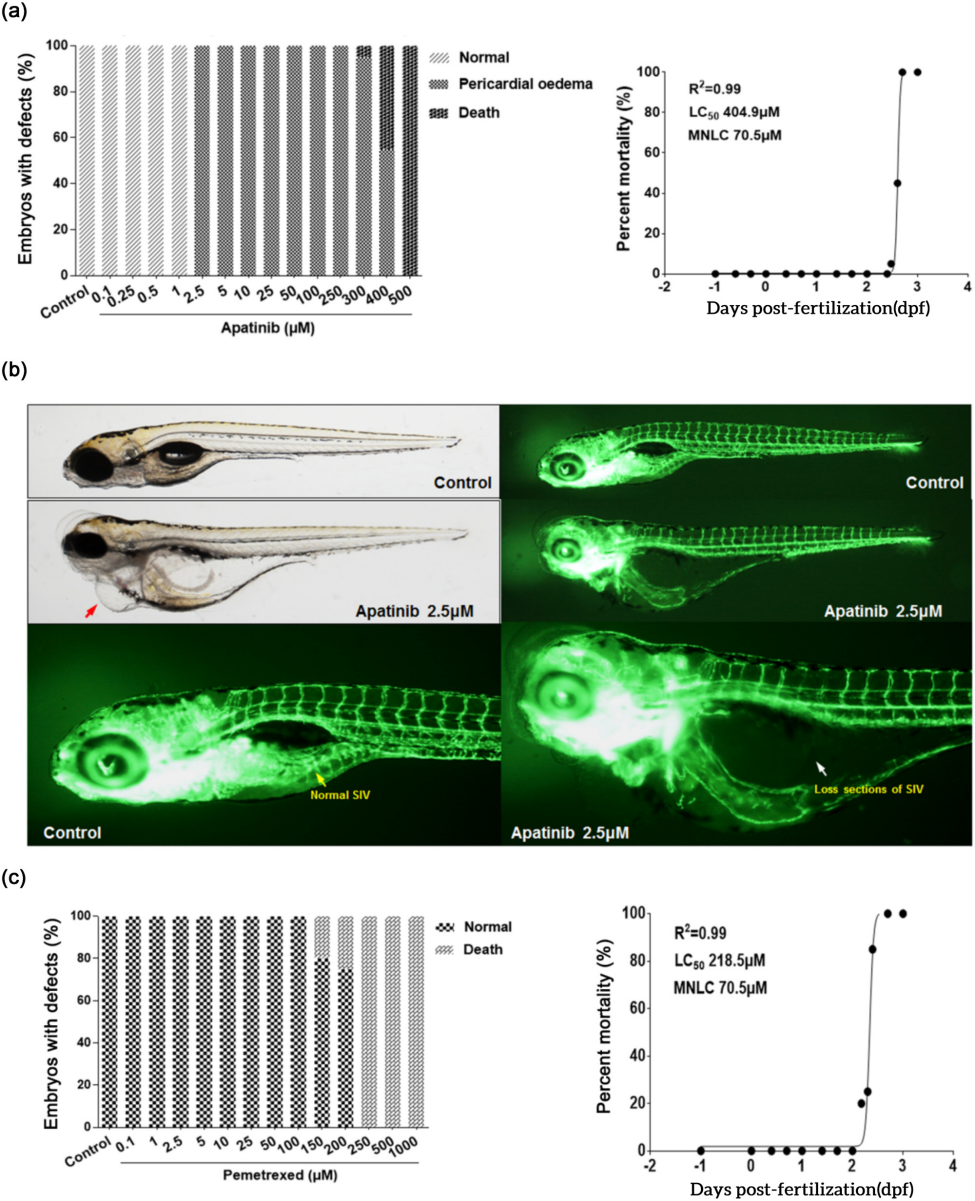
Toxicity and safety of apatinib and pemetrexed in the zebrafish model. (a) The number of animal situations observed after apatinib treatment. (b) Treatment with 2.5 μM apatinib for 48 h caused pericardial edema (red arrow) and heart failure in all zebrafish embryos. The complications were caused due to vascular defects in the SIV (white arrow). (c) The number of animal situations observed after pemetrexed treatment. Zebrafish Strain: Tg(fli1a:EGFP)y1. Route of Administration: Soaking in 0.1% DMSO (in fish water). Animal number: Total of 20 embryos for each condition.

The toxicity of apatinib and pemetrexed in zebrafish embryos was concentration-dependent. Compared with pemetrexed, apatinib was less toxic to zebrafish embryos, with an LC_50_ value of 404.9 μmol and MNLC value of 70.5 μmol (Figure 2a). In the experiment, treatment with 1 μmol apatinib revealed malformed zebrafish, but treatment with 2.5 μmol apatinib for 48 h caused pericardial edema and heart failure in all zebrafish embryos investigated under a microscope (Figure 2b). It is important to note that the complications that occurred here were due to the vascular defects in the subintestinal vein (SIV) rather than direct cardiac toxicity. Pemetrexed at LC_50_ value of 218.5 μmol and MNLC value of 70.5 μmol remained toxic to zebrafish embryos (Figure 2c). These results confirmed that the LC_50_ value of apatinib in zebrafish embryos was only half the value of pemetrexed, suggesting that apatinib is a low toxic agent.

### 3.3 Antiangiogenic effect of apatinib in the zebrafish model

**Figure 3:**
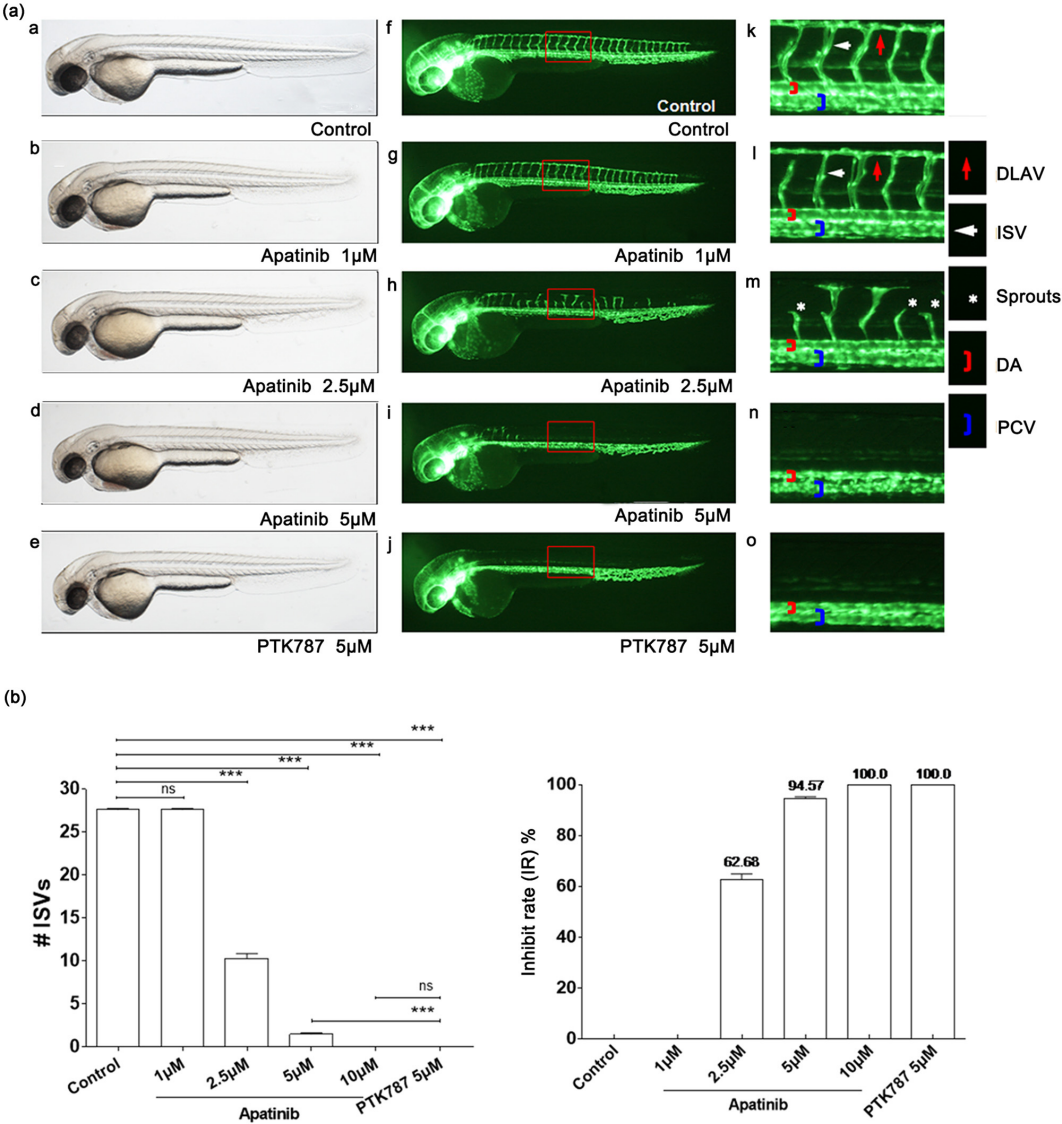
Apatinib inhibits angiogenesis in zebrafish in a dose-dependent manner. (a) (a–o) Representative bright field and fluorescent images of zebrafish embryos at 49 hpf treated with 0.1% DMSO control, apatinib (1, 2.5, 5, and 10 μM), or 5 μM PTK787 (positive control) for 26 h were observed. (f–o) Compared with controls, embryos treated with apatinib presented fewer incomplete ISVs and only occasional sprouts (asterisk) of DA. The boxed regions are shown at higher magnification in the right panels. (b) Quantification of the number of complete ISVs showed a significant decrease in the apatinib-treated embryos. The antiangiogenic effect of apatinib in zebrafish embryos was dose-dependent. # ISVs: The number of complete ISVs (the number of ISVs that connect the DA to the DLAV). Error bars, SEM; ***P < 0.0001 (*n* = 10; ANOVA); ns, not significant. DLAV, dorsal longitudinal anastomotic vessels; ISV, intersegmental vessels; DA, dorsal aorta; PCV, posterior cardinal vein. Zebrafish strain: fli1a-EGFP; casper. Route of administration: soaking in 0.1% DMSO (in fish water). Animal number: 30 embryos for each condition.

In order to assess the antiangiogenic property of apatinib *in vivo*, the inhibitory effects of apatinib on blood vessel development were assessed using a fli1a-EGFP and casper transgenic zebrafish model. The results demonstrated that apatinib inhibited angiogenesis in zebrafish in a dose-dependent manner and both apatinib and PTK787 [25] (positive control) dramatically inhibited the development of blood vessels compared with the vehicle-treated embryos, while the DA) and major cranial vessels were not inhibited (Figure 3a, Supplementary Material). Apatinib was administered at 1, 2.5, 5, and 10 μmol, and the inhibitory rates of ISV were 0, 62.68, 94.57, and 100%, respectively. When micro-injected with 10 μmol of apatinib, the number of ISVs was decreased to 0, similar to the PTK787 group (Figure 3b). These results suggested that apatinib strongly inhibited angiogenesis but did not affect the developed and mature vasculature.

### 3.4 Synergistic effect of apatinib combined with pemetrexed in the zebrafish A549 xenograft model

**Figure 4:**
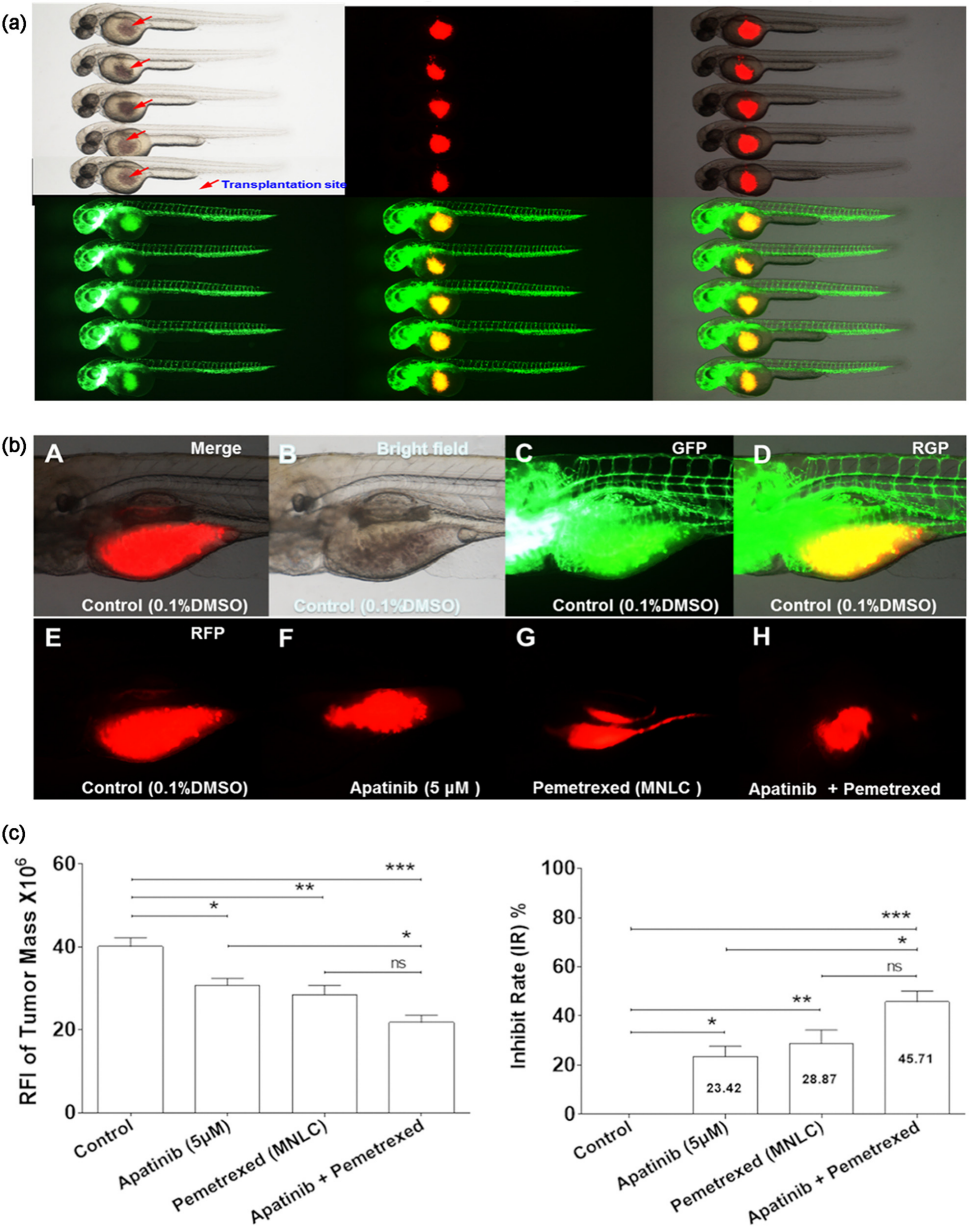
Co-administration of apatinib and pemetrexed inhibited tumor growth *in vivo*. (a) DiI-stained human A549 cells were successfully grafted into the yolk sac of a zebrafish embryo 2 dpf without immunosuppressant treatment. Approximately 200 cells were injected into the yolk sac and assessed by fluorescence microscopy. (b) Tumor growth at 4 dpi was observed by fluorescence microscopy. (c) Quantitative analysis of inhibitory effects of Apatinib, Pemetrexed, or combination concerning tumor growth. Columns, mean; bars, SEM (*n* = 10; ANOVA; ***P < 0.0001, **P < 0.01, *P < 0.05, compared to vehicle control group; *P < 0.05, compared to combination group). RFI, relative fluorescence intensity; dpi, days post-injection; MNLC, maximum non-lethal concentration. Zebrafish Strain: fli1a-EGFP; casper. Route of Administration: Soaking in 0.1% DMSO (in fish water).

A549 cells were stained with the red fluorescent cell tracer CM-Dil. A549 cells were microinjected into the yolk sac of the zebrafish embryo without immunosuppressant treatment. The tumor xenografts were evaluated by fluorescence microscopy immediately after injection (Figure 4a). The embryos with red fluorescence were moved to 6-well plates and cultured in fish water containing the drugs of interest (5 μmol apatinib with or without 70.5 μmol pemetrexed). The tumor growth on 4 dpi was evaluated by fluorescence microscopy, and the RFI was measured to estimate the volume of tumor xenografts in control and drug-treated animals (Figure 4b). The quantitative analysis of the inhibitory effects of apatinib, pemetrexed, or their combination on tumor growth showed that apatinib or pemetrexed alone or in combination significantly inhibited tumor growth in 4 dpi zebrafish embryos compared with the vehicle control group (Figure 4c). The inhibitory rates of the tumor mass in the apatinib, pemetrexed, and combination groups were 23.42, 28.87, and 45.71%, respectively (Figure 4c). These results revealed that apatinib had definite antitumor activity and suggested that the combination of apatinib and pemetrexed might be a potential alternative therapy for lung cancer.

### 3.5 Co-treatment of apatinib and pemetrexed synergistically inhibited VEGFR2, EFNB2A, ROBO4, and FGFR4

**Figure 5:**
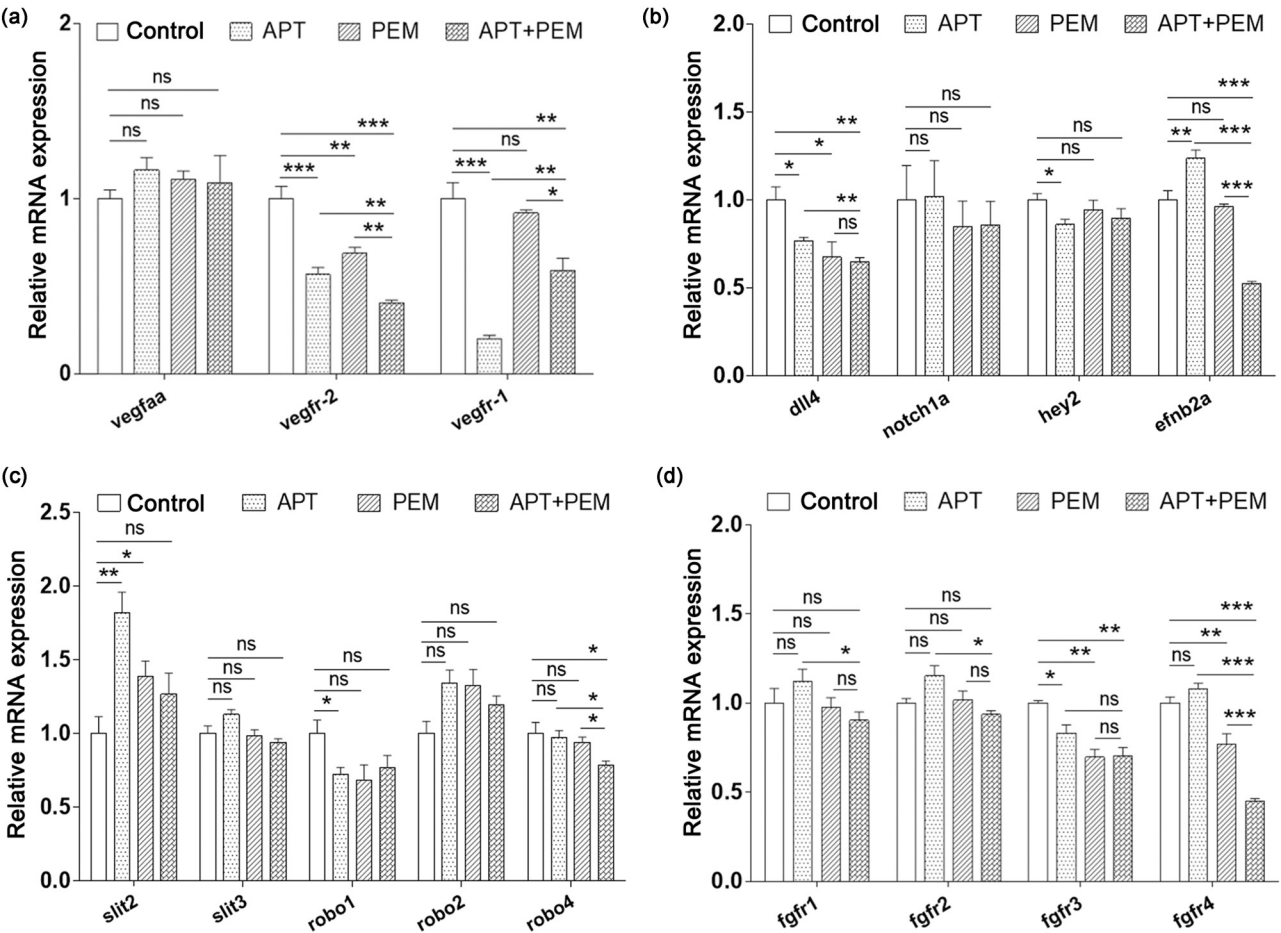
Effect of apatinib combined with pemetrexed on signaling pathways in zebrafish models. (a) Endogenous VEGFaa, VEGFR2, and VEGFR1 in control and lead compounds treated embryos were assessed by qRT-PCR (*n* = 10–15 individual embryos). APT, apatinib; PEM, pemetrexed; ns, not significant. (b) Endogenous Dll4, Notch1a, Hey2, and EFNB2A in control and lead compounds treated embryos were assessed by qRT-PCR (*n* = 10–15 individual embryos). (c) Endogenous SLIT2, SLIT3, ROBO1, ROBO2, and ROBO4 in control and lead compounds treated embryos were assessed by qRT-PCR (*n* = 10–15 individual embryos). (d) Endogenous FGFR11, FGFR 2, FGFR 3, and FGFR 4 in control and lead compounds treated embryos were assessed by qRT-PCR (*n* = 10–15 individual embryos). Zebrafish strain: Tg (fli1a:EGFP)y1. Animal number: 90 embryos for each condition.

In order to identify the molecular mechanism of the combinatorial effect of apatinib and pemetrexed in zebrafish, the mRNA expression levels of VEGFaa-VEGFR, Dll4-Notch-Hey2, EFNB2a, SLIT-ROBO, FGFR, PTP-RB, COX-2, VE-cadherin, and PIK3R2 were determined in the different treatment groups, which were the same as the above zebrafish A549 xenograft experiments. As shown in Figure 5a, no remarkable inhibition of the mRNA level of VEGFaa was observed when treated with single-agent or combined treatments. The mRNA expression levels of VEGFR1 (*P <* 0.0001) and VEGFR2 (*P <* 0.0001) were suppressed by apatinib treatment. Pemetrexed also suppressed the mRNA level of VEGFR2 (*P <* 0.01) but did not affect VEGFR1. The results revealed that the mRNA level of VEGFR2 in the co-treatment group was significantly decreased when compared to either the vehicle control group (*P <* 0.0001) or the single-agent group (*P <* 0.01), suggesting that apatinib and pemetrexed synergistically inhibited the VEGFR2 pathway.

Apatinib or pemetrexed reduced the mRNA expression level of Dll4 when compared with the vehicle control group (*P <* 0.05), while apatinib combined with pemetrexed significantly inhibited the expression of Dll4 when compared with apatinib and control groups (*P <* 0.01), (Figure 5b).

The inhibition of VEGFR2 expression with apatinib induced a compensatory upregulation of the EFNB2A gene in zebrafish (*P <* 0.01). Pemetrexed did not affect the expression of EFNB2A compared with the vehicle control group, while the combination of apatinib and pemetrexed significantly inhibited the mRNA levels of EFNB2A (*P <* 0.0001), suggesting that apatinib and pemetrexed synergistically inhibited EFNB2A pathway (Figure 5b).

In this experiment, no remarkable inhibition of the mRNA levels of SLIT3, ROBO1, and ROBO2 was observed in the different treatment groups. The SLIT2 gene expression induced compensatory upregulation in zebrafish embryos treated with apatinib or pemetrexed. Zebrafish embryos, when co-treated with apatinib and pemetrexed, demonstrated suppression of SLIT2 mRNA expression upregulation to some extent but showed no significant difference between the combination group and vehicle group. For ROBO4, both single-agent groups demonstrated no effect on its expression level. However, the mRNA expression level of ROBO4 was significantly reduced in the combination group (*P <* 0.05), suggesting that apatinib and pemetrexed synergistically inhibited the ROBO4 pathway (Figure 5c).

The expression levels of FGFR1 and FGFR2 showed no significant differences among the four groups. Both apatinib and pemetrexed inhibited the expression of FGFR3, but no synergistic effect was observed. As for FGFR4, apatinib showed no effect on its expression, but pemetrexed inhibited its expression (*P <* 0.01). In addition, the combination treatment significantly inhibited the expression of FGFR4 (*P <* 0.0001), suggesting that apatinib and pemetrexed synergistically inhibited the FGFR4 pathway (Figure 5d).

In addition, though PTP-RB, COX-2, VE-cadherin, and PIK3R2 are associated with angiogenesis [26,27,28,29], no remarkable changes in their mRNA levels were observed when treated with single-agents or combined agents (Supplementary Material).

These findings suggested that apatinib combined with pemetrexed had enhanced antitumor effects compared with either one alone in a zebrafish model of NSCLC, which involves the synergistic inhibition of the VEGFR2, EFNB2A, ROBO4, and FGFR4 signaling pathways.

## 4 Discussion

In this study, we objectively evaluated the combined effects of apatinib and three first-line chemotherapy agents against NSCLC both *in vitro* and *in vivo*. In the *in vitro* experiment, apatinib demonstrated a slight inhibitory effect on the viability of A549 lung cancer cells, but compared with cytotoxic agents, the IC_50_ value remained higher, suggesting it was a low toxic agent. Apatinib-based chemotherapy strategy had a greater antitumor effect than single-agent treatments. Apatinib combined with pemetrexed had more optimal antitumor effects when compared with gemcitabine or paclitaxel *in vitro*, suggesting that apatinib and pemetrexed might be an optimal combination treatment.

In the *in vivo* experiments, the LC_50_ value of apatinib in zebrafish embryos was only half the value of pemetrexed, suggesting that apatinib is a low toxic and highly safe agent. The inhibitory effects of apatinib on blood vessel development were examined using a transgenic zebrafish model. The 22 hpf embryos were treated with different concentrations of apatinib for 26 h. Apatinib strongly inhibited the formation of ISVs but did not affect the developed DAs, suggesting that apatinib can effectively inhibit angiogenesis in tumors but have little effect on the normal vasculature. It demonstrated the high safety of apatinib.

Considering that the combination of apatinib and pemetrexed can be used to induce an optimal anticancer effect *in vitro*, the antitumor effect of apatinib combined with pemetrexed was evaluated in the zebrafish A549 xenograft model. Apatinib and pemetrexed, whether alone or in combination, significantly inhibited tumor growth, and their combination yielded the most optimal antitumor effect. It suggested that the combination of apatinib and pemetrexed might be a promising alternative therapy in patients with lung cancer. A study showed that anlotinib plus pemetrexed could be used for platinum-resistant ovarian cancer [30]. Another study also suggested the combination of anlotinib, pemetrexed, and cisplatin as first-line therapy for advanced NSCLC [31]. Although only a few data are available regarding the clinical use of anlotinib plus pemetrexed, the present study supports using this combination in lung cancer. Future studies are necessary to determine the exact benefits and risks of that combination.

The expression of Dll4 was dependent on continuous VEGF signaling. Blockage of the VEGF signaling pathway causes a rapid and marked decrease in the expression of Dll4 by tumor vessels. The *Ephrin-B2* gene is the equivalent of the EFNB2A gene in zebrafish. Blocking *Ephrin-B2* reverses VEGFR2 signaling and is considered an attractive alternative or combinatorial antiangiogenic therapy strategy for disrupting VEGFR2 function in tumor angiogenesis [18]. SLIT2 promoted the migration of endothelial cells and promoted the angiogenic activity via the ROBO1-VEGFR2-ERK1/2 signaling pathway; current data showed that the SLIT/ROBO pathway could be a promising therapeutic target for cancer [19,20]. FGFR signaling affects vascular outgrowth and is required to maintain blood vessel integrity by ensuring proper cell-cell junctions between endothelial cells. VEGFR inhibitor treatment caused compensatory upregulation of the FGFR pathway, contributing to tumor relapse [21]. The *VE-PTP* gene is equivalent to the *ptp-rb* gene in zebrafish, wherein it regulates VEGFR2 activity in stalk cells to establish endothelial cell polarity and lumen formation [26]. Moreover, *COX-2* and *PIK3R2* genes are also associated with tumor angiogenesis [27,28,29].

In our study, we initially found that apatinib directly inhibited the expression of VEGFR2, and pemetrexed significantly enhanced this inhibitory effect in the zebrafish A549 xenograft model. In addition, due to the antiangiogenic treatment of the zebrafish A549 xenograft model, compensatory upregulation of EFNB2A and SLIT might be associated with antiangiogenic drug resistance and tumor relapse. Surprisingly, when zebrafish embryos were treated with apatinib and pemetrexed, the compensatory upregulation of SLIT2 and EFNB2A mRNA expression showed suppression to some extent. It suggested that apatinib combined with pemetrexed might delay antiangiogenic drug resistance. Finally, apatinib and pemetrexed co-treatment also synergistically inhibited FGFR gene expression, which may enhance the antiangiogenic effect of the apatinib single-agent. Therefore, apatinib combined with pemetrexed has more advantages compared with single-agent treatment.

So far, the known side effects of apatinib mainly include hypertension (69.5%), proteinuria (47.8%), and hand-foot syndrome (45.6%), and some patients have to stop apatinib even though they have benefited from it when intolerance to adverse events occur [32]. Therefore, the tolerance regarding the combination of apatinib and pemetrexed is also an important point that needs to be examined. Our data showed that the combination of apatinib with pemetrexed did not significantly lead to malformation or acute mortality of zebrafish when compared with other single-agents *in vivo*. In this study, pemetrexed potentiated the effects of apatinib both *in vitro* and *in vivo*, making it possible to reduce the dosage of current apatinib and cytotoxic agents by using a drug combination regimen.

We agree that rodent models are closer to humans than zebrafish, but we needed a model that allowed the evaluation of the antiangiogenic effect of apatinib during development and at vessel maturity. In addition, zebrafishes can be used as xenograft models for human cancer cells. In this case, the drugs were tested on the tumors from the A549 cells, not on the zebrafish. Nevertheless, future studies will be performed on rodents [33,34,35].

This study has provided a strong rationale to combine apatinib with current chemotherapeutic agents for treating advanced NSCLC in patients who do not harbor identifiable driver oncogenes. We believe that the combination of apatinib and pemetrexed is a promising therapeutic strategy for patients with advanced NSCLC.

## Supplementary Material

Supplementary material
