# Supplementary material for: The apatinib and pemetrexed combination has antitumor and antiangiogenic effects against NSCLC
Source: Open Life Sci. 2023 Mar 7;18(1):20220533. doi: 10.1515/biol-2022-0533 (PMC9993329; doi:10.1515/biol-2022-0533)
Supplement: Supplementary material [file biol-2022-0533-sm.pdf]

Supplementary Materials

See Tables S1–S3 and Figures S1–S4 in the Supplementary Material for expanded data.

Table S1: The cell viability of A549 cells in different combination modes

A concentration (μM)	B1 concentration (μM)	A549 cell viability %		
5		48.48066	50.13812	48.75691
1.592356688		71.54696	83.01105	82.18232
0.507119964		83.28729	83.56354	92.12707
0.161503173		93.09392	94.47514	95.58011
0.051434132		100.6906	98.34254	95.99448
4	1	26.40693	24.5671	32.35931
1.27388535	0.318471338	48.16017	44.91342	52.38095
0.405695971	0.101423993	61.03896	70.99567	64.06926
0.129202539	0.032300635	71.9697	73.59307	66.01732
0.041147305	0.010286826	75	73.91775	72.94372
3	2	33.76623	34.30736	39.17749
0.955414013	0.636942675	52.70563	50.64935	53.24675
0.304271979	0.202847986	66.77489	61.90476	67.74892
0.096901904	0.064601269	83.00866	84.19913	75.75758
0.030860479	0.020573653	77.81385	84.74026	82.57576
2	3	19.95321	25.31994	25.45755
0.636942675	0.955414013	58.34595	53.66726	55.73139
0.202847986	0.304271979	74.0333	62.19898	61.64855
0.064601269	0.096901904	91.78478	96.87629	93.84891
0.020573653	0.030860479	101.555	101.1421	99.62846
1	4	18.30191	21.05408	16.92583
0.318471338	1.27388535	42.24577	43.48424	41.1449
0.101423993	0.405695971	49.53901	48.02532	50.36466
0.032300635	0.129202539	78.71199	74.0333	68.11614
0.010286826	0.041147305	79.26242	80.22568	76.37264
	5	38.25513	37.56708	41.97055
	1.592356688	46.78684	45.27315	44.58511
	0.507119964	51.74075	44.17229	48.98858
	0.161503173	55.59378	56.41943	57.65791
	0.051434132	71.14353	67.70332	79.12481
A concentration (μM)	B2 concentration(nM)	A549 cell viability %		
5		46.56284	47.50669	43.41671
1.592356688		79.91191	78.18153	76.60846

(*Continued*)

Table S1: *Continued*

A concentration (μM)	B1 concentration (μM)	A549 cell viability %		
0.507119964		84.15919	81.64228	82.58613
0.161503173		91.23801	89.35032	95.6426
0.051434132		100.6764	97.68759	99.57527
4	0.4	36.4952	30.98946	26.27025
1.27388535	0.127388535	65.43967	55.84395	50.49552
0.405695971	0.040569597	77.08038	68.58581	61.66431
0.129202539	0.012920254	98.15951	87.30533	78.81076
0.041147305	0.004114731	103.1933	98.0022	97.05836
3	0.8	48.45053	35.39405	38.54019
0.955414013	0.25477707	58.67548	58.83278	51.75397
0.304271979	0.081139194	88.40648	76.45116	81.17036
0.096901904	0.025840508	101.3056	97.8449	96.90105
0.030860479	0.008229461	103.1933	97.37297	98.0022
2	1.2	23.2364	10.71379	24.34952
0.636942675	0.382165605	40.62891	29.21942	30.1934
0.202847986	0.121708791	78.47502	63.03047	78.61416
0.064601269	0.038760762	101.9897	97.25894	99.9026
0.020573653	0.012344192	97.67636	101.294	100.7374
1	1.6	19.0622	11.54863	3.061083
0.318471338	0.50955414	32.69793	30.05426	38.68095
0.101423993	0.162278389	87.93655	78.33588	92.11076
0.032300635	0.051681015	98.92862	101.4331	99.06776
0.010286826	0.016458922	98.5112	98.92862	102.268
	2	15.72283	21.01016	19.47962
	0.636942675	33.81105	28.94114	34.64589
	0.202847986	58.16057	63.44789	61.63907
	0.064601269	85.15375	85.57117	90.44107
	0.020573653	95.7284	97.8155	100.8766
5		37.59515	53.9071	46.45021
1.592356688		82.18114	86.37564	86.53099
0.507119964		80.93833	97.09492	92.43436
0.161503173		93.52183	99.58055	99.89125
0.051434132		95.2307	101.9108	83.57931
4	1	34.33276	29.51686	32.00249
1.27388535	0.318471338	54.99456	52.81964	59.65512
0.405695971	0.101423993	57.01414	62.76216	60.27653
0.129202539	0.032300635	61.36399	70.52975	65.55849
0.041147305	0.010286826	85.59888	75.50101	111.8533
3	2	24.5456	34.02206	31.38108
0.955414013	0.636942675	38.52726	42.72176	46.91627

(*Continued*)

Table S1: *Continued*

A concentration (μM)	B1 concentration (μM)	A549 cell viability %		
0.304271979	0.202847986	51.26612	45.5181	50.48936
0.096901904	0.064601269	70.06369	61.36399	76.43312
0.030860479	0.020573653	69.75299	76.58847	68.97623
2	3	40.43698	34.5671	38.96951
0.636942675	0.955414013	50.38317	52.66591	55.92695
0.202847986	0.304271979	56.41611	58.53579	58.69884
0.064601269	0.096901904	62.77515	66.19925	64.56873
0.020573653	0.030860479	81.68922	82.66754	79.08038
1	4	42.0675	35.70846	30.81689
0.318471338	1.27388535	44.51329	49.89402	67.34062
0.101423993	0.405695971	56.74221	51.03538	72.06913
0.032300635	0.129202539	53.31812	70.60166	75.33018
0.010286826	0.041147305	64.56873	74.84102	67.82977
	5	33.58878	33.91489	30.97994
	1.592356688	49.73096	48.5896	45.1655
	0.507119964	54.62253	52.3398	58.04663
	0.161503173	58.37274	57.23137	60.65547
	0.051434132	80.87396	77.12376	79.40649

Table S2: IC50 value of Apatinib and chemotherapy agent in different treatment groups

A combined with B	A+B1		A+B2		A+B3	
	Drug A(μM)	Drug B1(μM)	Drug A(μM)	Drug B2(nM)	Drug A(μM)	Drug B3(μM)
A	5.384	0	2.693	0	5.275	0
B	0	0.75	0	0.484	0	0.876
4/5A+1/5B	0.827	0.207	1.43	0.143	1.1	0.274
3/5A+1/5B	1.09	0.728	1.568	0.418	0.442	0.295
2/5A+3/5B	0.644	0.966	0.641	0.385	0.579	0.868
1/5A+4/5B	0.12	0.483	0.313	0.501	0.286	1.143

Table S3: qRT-PCR primers for Zebrafish

Gene	Forward primer 5′–3′	Reverse primer 5′–3′
vegfaa	CTCCATCTGTCTGCTGTAAAGG	GGGATACTCCTGGATGATGTCTA
kdr/flk1/ vegfr	CCTGAGACCATCTTTGACCG	GTTCCCTCTT TAAGTCGCCTG
ft1/vegfr-1	GTATTTGAACAGCACGGGTTTAG	CGGCTTCTTGATATGCGTTTG
Dll4	AGGCCTGGCACTCACCTTACTC	CACCCCAGCCCTCTTTACAGTT
Notch1a	GCCGCAGATGCAGGGCAATGAAGT	GAGGGCAGGCAGGGCTGGTAGAGG
hey2	CGGCTTCCGGGAGTGTCTGACT	TCCCCACGGTCGGTATGGTTTA
efnb2a	TTGGGGCCTGGAGTTCTTCAGA	TCTTGGGCGTGGCTAATGTGCT
Slit2	GAGCGACTGGACCTGAATG	GTAGATCCTGAAATGCCCCTC
slit3	GCGAGTGTTTCCAAGACCTG	GATTTCATTGTCGTTCAGCCG
Robo1	AGGAGTCACATACAGGCTAGAG	GTCTGAGATCTGCTGGGAAATG
Robo2	GAGGTGTGGATGTGGACTATG	CTACAATCCGAGGTGGAGAATC
robo4	GAGATCAGTCCCAAACCACAG	CCCACAGATATAGCCCAACG
Fgfr1	CCTGCGCAACGATCAGATGGAGAT	TTGCCGTTTTTAAGCCACTTGAGC
Fgfr2	CCCCGACAACCGCACGCTCGTA	TAGCCGCCCATGCGATCCTCCTGT
Fgfr3	TCCCCGTATCCAGGTATCC	TCTGAACGTGGGTCTTTGTG
Fgfr4	GCTGCGCATTGGATTGACCGATAA	AGGCCCCAGGCGATTACTGTCCTT
COX2	CCTTCCGGCCATCATTCTTATT	CCGCAGATTTCAGAGCATTGTC
Mmp9	GTGCCGGACATCCGCAACTACA	CACCAGGGAAGGCCATCTGTGC
VE-cadherin/cdh5	TGCTCGCATTATGTAGCCTCAGTA	AGAATCGAAAACGGGGATGTTG
ptp-rb	TTGGGCAGCATGCGGAATACTGAG	TTACCAGGCTGCCATGAAACATCC
pik3r2/p85β	CCCGGAAACTGCTCCCCCTAATCT	AGCGGAGGAGTCGGCTCTTGTT
ef1	GGAAATTCGAGACCAGCAAATAC	GATACCAGCCTCAAACTCACC

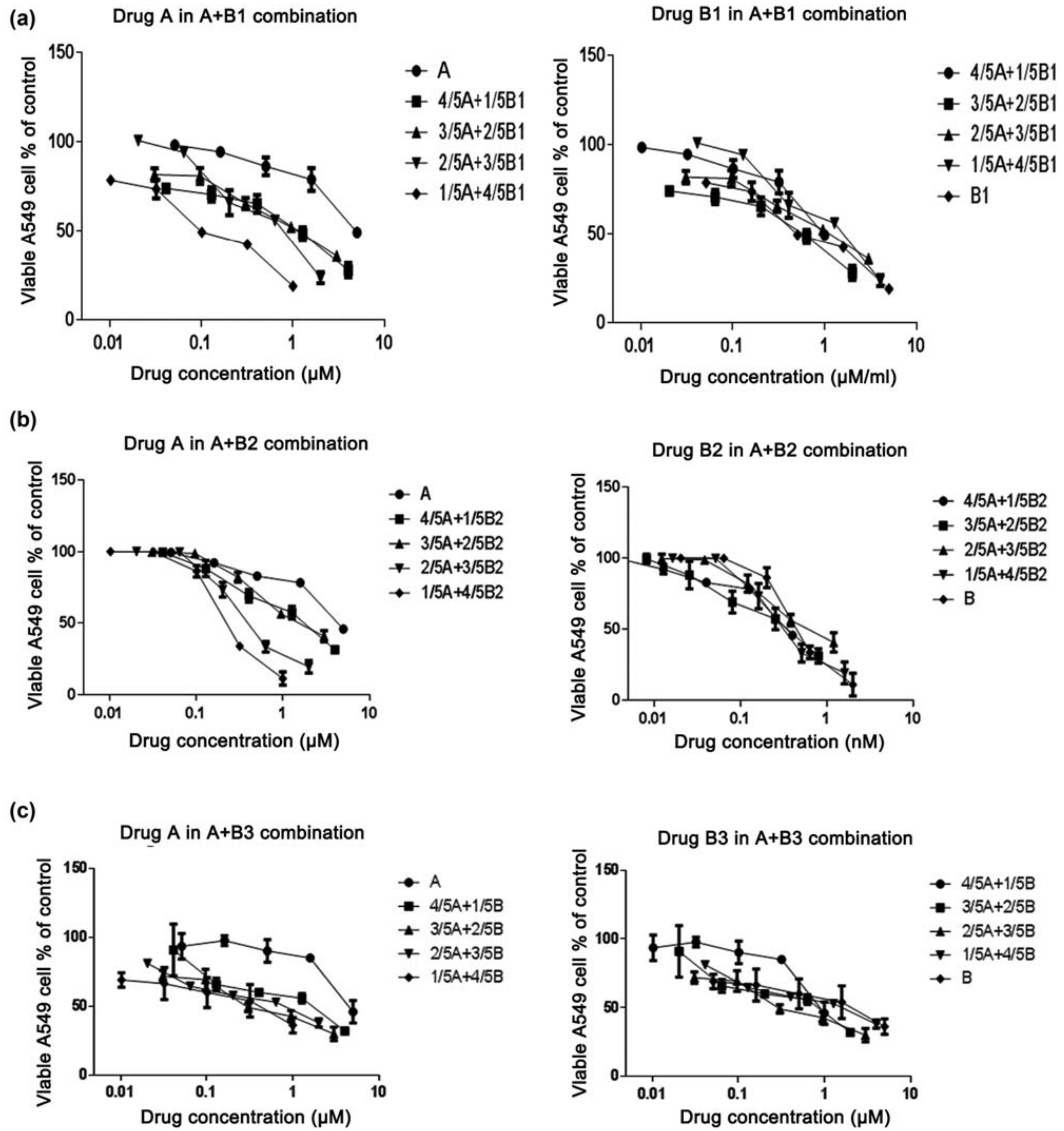

Figure S1: Effects of Apatinib combined with chemotherapy in A549 cell line. Apatinib was named drug A and cytotoxic agents were named drug B (Gemcitabine, Paclitaxel, Pemetrexed, were named B1, B2, B3, respectively) for the convenience of discussion. There are four combination proportions of Apatinib and cytotoxic agents. The anticancer effects of Apatinib/chemotherapy agent alone and Apatinib combined with chemotherapy agent were detected using MTT assay (S1A), (S1B), (S1C). As the drug concentrations of either Apatinib or Gemcitabine increased, the viability of A549 cell decreased.

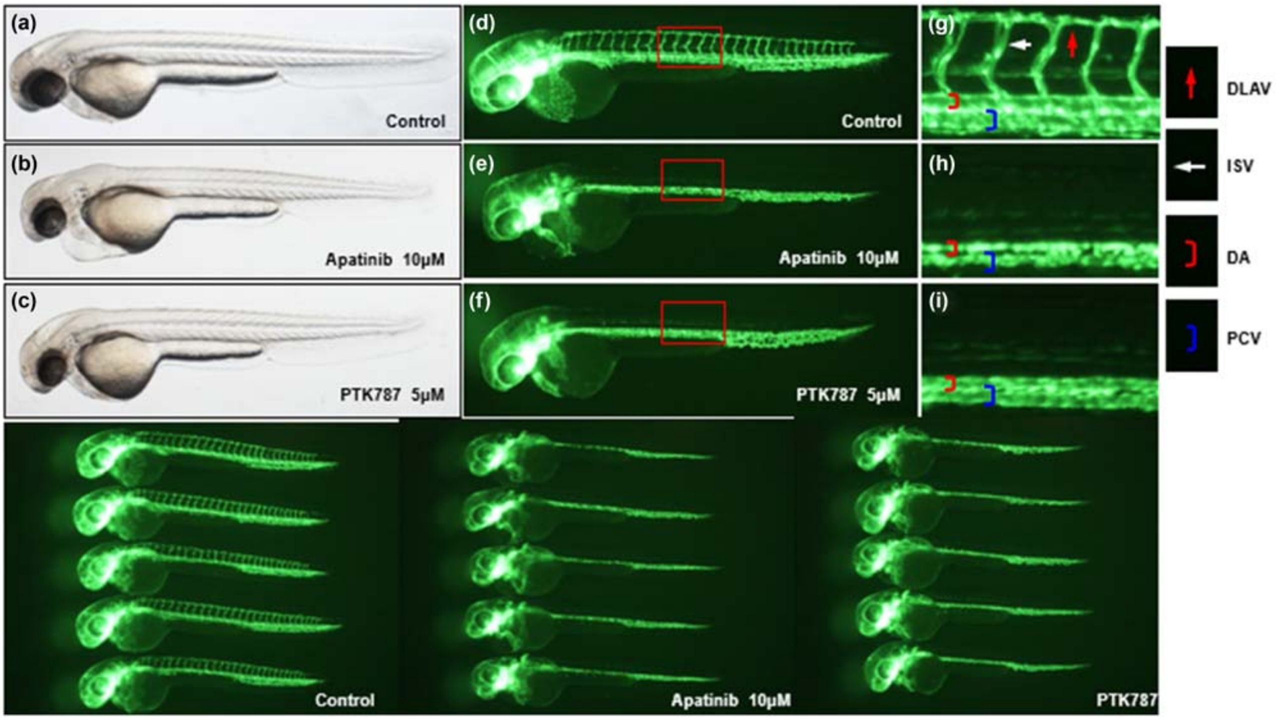

Figure S2: Apatinib inhibits zebrafish angiogenesis.

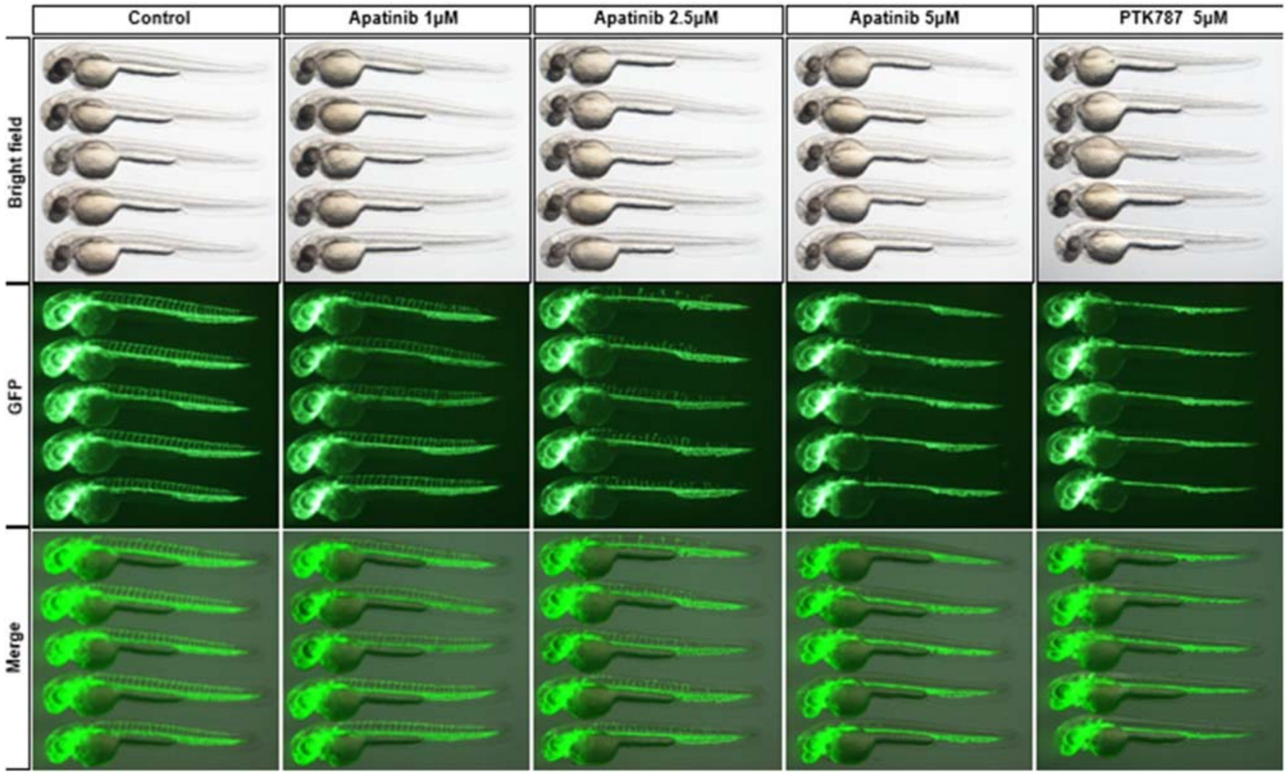

Figure S3: Apatinib inhibits zebrafish angiogenesis.

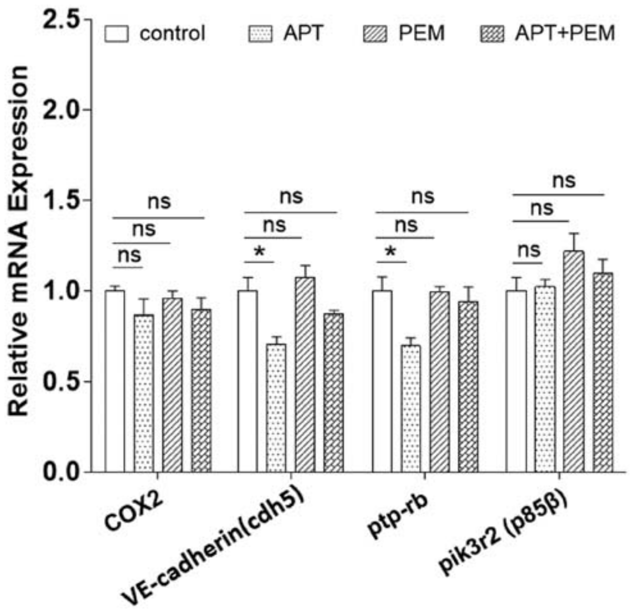

Figure S4: The relative mRNA expression levels of COX-2, VE-cadherin, PTP-RB and PIK3R2 genes in different treatment groups. (Apatinib, Pemetrexed and Apatinib combined with Pemetrexed were named APT, PEM, APT+PEM, respectively).
